# Current Rectification in a Structure: ReSe_2_/Au Contacts on Both Sides of ReSe_2_

**DOI:** 10.1186/s11671-018-2843-4

**Published:** 2019-01-03

**Authors:** Tingting Miao, Dongwei Yu, Lei Xing, Dawei Li, Liying Jiao, Weigang Ma, Xing Zhang

**Affiliations:** 1 Beijing Key Laboratory of Process Fluid Filtration and Separation, College of Mechanical and Transportation Engineering, China University of Petroleum-Beijing, Beijing, 102249 China; 2 Key Laboratory for Thermal Science and Power Engineering of Ministry of Education, Department of Engineering Mechanics, Tsinghua University, Beijing, 100084 China; 3 Department of Chemistry, Tsinghua University, Beijing, 100084 China

**Keywords:** ReSe_2_, Rectification, Two-dimensional materials

## Abstract

Schottky effect of two-dimensional materials is important for nanoscale electrics. A ReSe_2_ flake is transferred to be suspended between an Au sink and an Au nanofilm. This device is initially designed to measure the transport properties of the ReSe_2_ flake. However, a rectification behavior is observed in the experiment from 273 to 340 K. The rectification coefficient is about 10. The microstructure and elements composition are systematically analyzed. The ReSe_2_ flake and the Au film are found to be in contact with the Si substrate from the scanning electron microscope image in slant view of 45°. The ReSe_2_/Si and Si/Au contacts are p-n heterojunction and Schottky contacts. Asymmetry of both contacts results in the rectification behavior. The prediction based on the thermionic emission theory agrees well with experimental data.

## Introduction

Rectification behaviors of metal-semiconductor contacts, where the current varies with the direction of the applied voltage, are widely used in Schottky barrier diode, field effect transistor (FET), and metal-oxide-semiconductor FET. Schottky explained the behavior by depletion layers on the semiconductor side of such interfaces [1]. Differences of electron work function between metal and semiconductor lead to the rectification behavior named Schottky effect [2]. The contact between metal and two-dimensional (2D) semiconductor materials is a Schottky contact when the metal has a higher electron work function than an n-type 2D semiconductor materials or lower electron work function than a p-type 2D semiconductor. The Schottky effect of metal/2D materials has great applications in micro-photo detectors, micro-FETs, gas sensors, and phototransistors [3]. Among 2D materials, transition metal dichalcogenides (TMDs) have attracted much attention because they have a sizable bandgap [3] and the bandgap transits from indirect to direct as the thickness is reduced to monolayer [4]. The bandgap ensures that TMDs can be used for many applications, i.e., FETs and solar cells [3]. TMDs can be also used in thermoelectric field [5], which has drawn wide attention [6–9]. Many experiments have been done to explore properties and applications of TMDs such as MoS_2_, MoSe_2_, WSe_2_, and WS_2_. Lopez-Sanchez et al. [10] made ultrasensitive monolayer phototransistors with MoS_2_. Britnell et al. [11] made a WS_2_/graphene heterostructure and demonstrated its application in photovoltaic device. WSe_2_, as an ambipolar semiconductor, was controlled with double electrostatic gates to fabricate a light-emitting diode [12, 13]. Among TMDs, ReSe_2_ is different from other group VI TMDs because ReSe_2_ belongs to group VII TMDs with an extra electron in *d* orbitals, which leads to strong in-plane anisotropy [14]. A few studies have explored the electrical properties of ReSe_2_ due to its special band structure. Current rectification is explored with a ReSe_2_/WS_2_ p-n heterojunction [15] and ReSe_2_/MoS_2_ p-n heterojunction [16]. FET is made to investigate the electrical properties of metal/semiconductor contacts like ReSe_2_/metal or ReS_2_/metal [17–19].

In this letter, a ReSe_2_ flake is suspended across an Au sink and an Au nanoribbon electrode. The device is originally designed to measure the thermal and electrical conductivities of the ReSe_2_ flake. Measurements were performed at 340 K, 310 K, 280 K, and 273 K.

## Methods

Firstly, the Si substrate with Au electrodes was fabricated. The 400-μm-thick undoped Si substrate was oxidized to form a 180-nm-thick SiO_2_ layer after initial cleaning, and a 320-nm-thick electron beam resist was deposited on the SiO_2_ surface by means of spin coating. Au was deposited by physical vapor deposition to fabricate the Au nano-electrodes and the Au nanofilm in the pattern which was prepared by electron beam lithography. By putting the sample into the photoresist developer, the electron beam resist was etched and the Au electrode and film were left. At last, the SiO_2_ layer is etched by buffered hydrofluoric acid and the Si layer under the Au nanofilm is etched by CF_4_ plasma to fabricate a suspended nanofilm which is about 6 μm above the Si substrate.

**Fig. 1:**
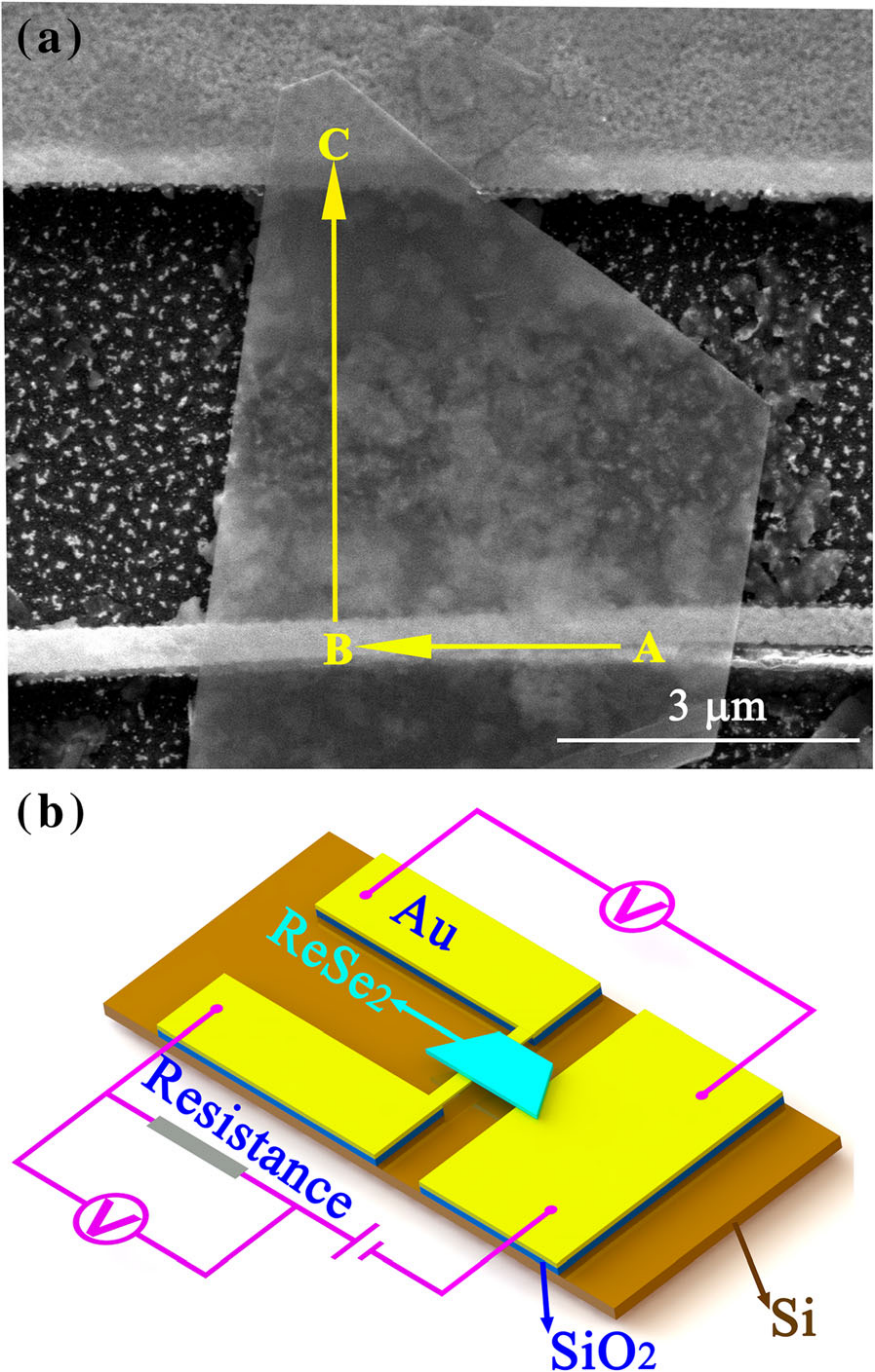
**a** SEM image of the device in vertical view to the substrate and the positive current direction and **b** schematic diagram of the measurement device

ReSe_2_ flakes were synthesized by chemical-vapor-transition on a copper substrate. A ReSe_2_ flake was transferred to the Au electrodes to fabricate Au-ReSe_2_-Au contacts using the wetting transfer method, in which the ReSe_2_ nanoribbon with the copper substrate was coated by polymethylmethacrylate (PMMA) and floated onto the etching solution to etch the copper substrate. After the copper substrate was peeled off, the PPMA-coated ReSe_2_ flake was accurately moved above the Si substrate with Au nano-electrodes by the fixed-point transfer platform. Then, the PMMA was cut by laser and the PMMA-coated ReSe_2_ flake landed to be suspended between the Au nanofilm and the Au nano-electrode. Finally, the PMMA was removed by dipping the sample into a potassium hydroxide solution bath for 3 h. The scanning electron microscope (SEM) image of the fabricated Au electrode-ReSe_2_ flake-Au nanoribbon (Au-ReSe_2_-Au) junctions in vertical view to the substrate is shown in Fig. 1a. The ReSe_2_ flake was in contact with an Au nanoribbon in section B and in contact with Au electrode in section C. Figure 1b shows the schematic diagram of the device.

The direction along A-B-C is defined as positive, or vice versa, and a direct current was applied. The voltage, *V*, across the Au-ReSe_2_-Au junctions was measured by a high accuracy digital multimeter (Keitheley 2002, 8.5 digits), while the current, *I*, was determined through measuring the voltage across a reference resistor in series. The *I*-*V* curves of the ReSe_2_/Au junctions for forward and inverse voltage were measured at different temperatures in a physical property measurement system (quantum design).

## Results and Discussion

**Fig. 2:**
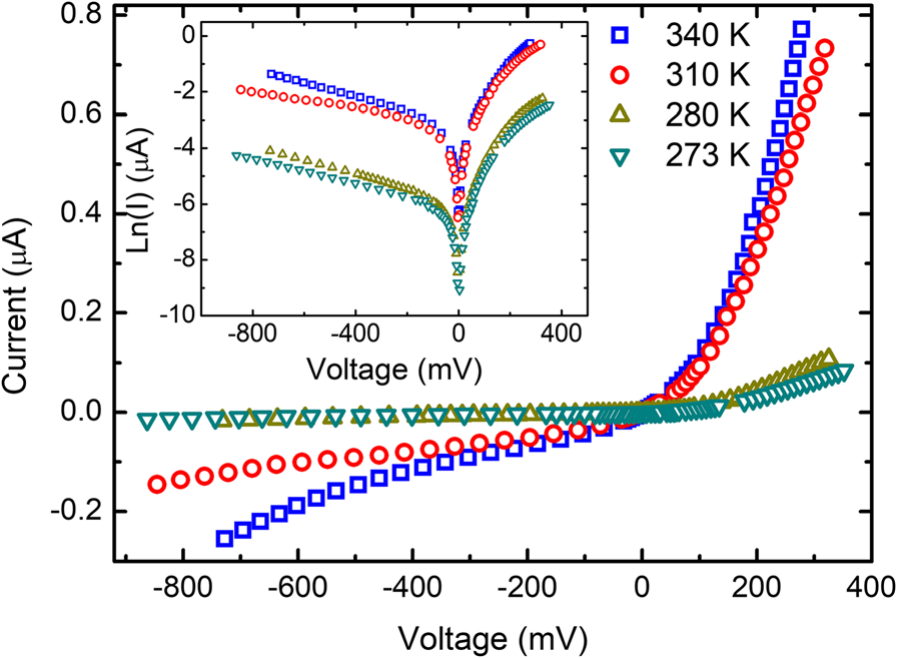
Current-voltage characteristics of Au-ReSe2-Au junctions at 273 K, 280 K, 310 K, and 340 K

Figure 2 shows the measured *I*-*V* curves at 273 K, 280 K, 310 K, and 340 K. Significant asymmetries in the *I*-*V* curves are observed at all the measured temperatures, indicating unusual rectifying behavior. Currents at 277 mV and − 277 mV are used to calculate the current rectification ratio at each temperature, and the rectifying ratio is about 10. The current increases with the temperature for a given voltage.

**Fig. 3:**
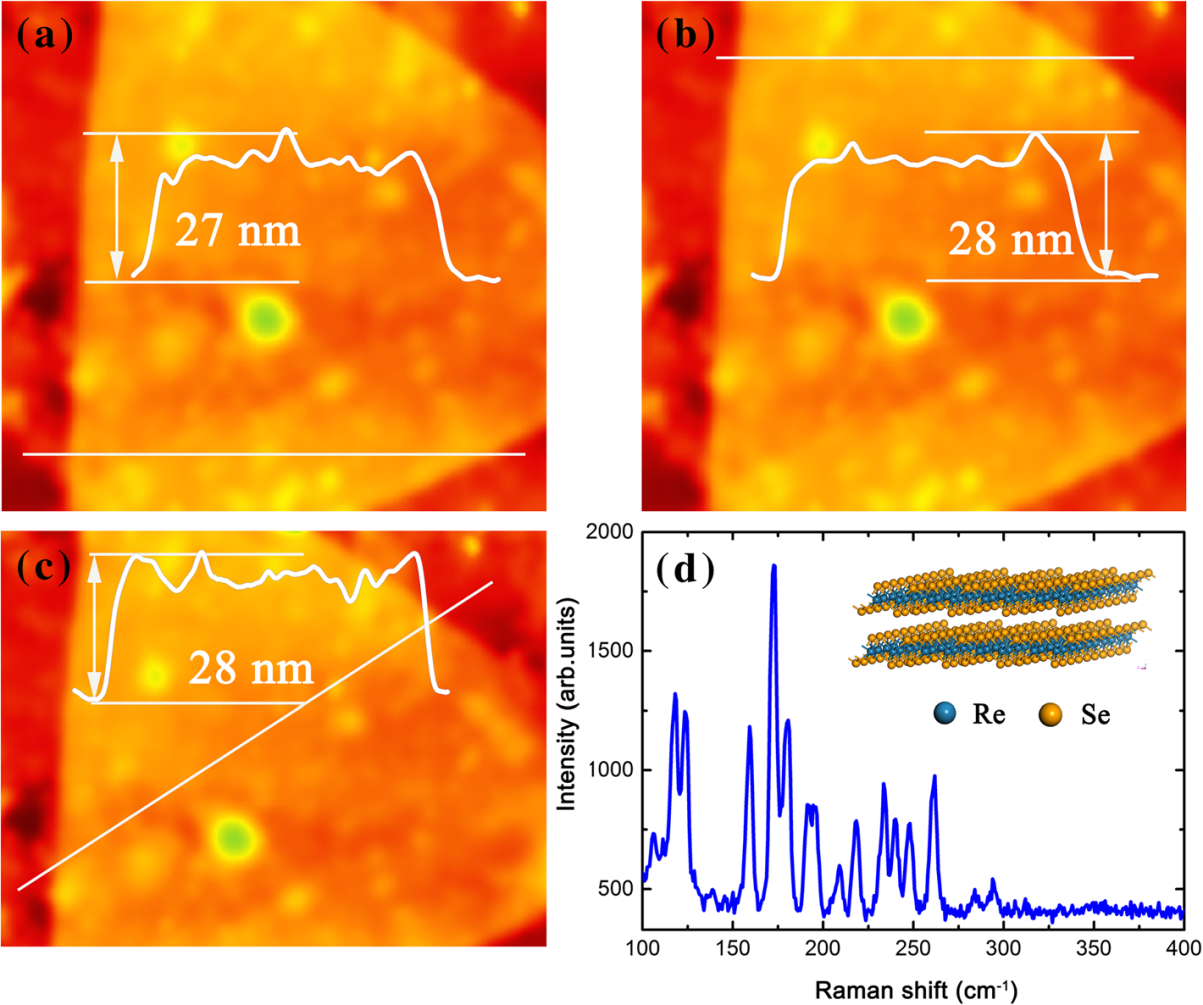
**a**, **b**, and **c** AFM image and thickness of ReSe2, and **d** Raman spectrum and crystal structure of ReSe2

To explore the mechanism responsible for the unusual rectification, the microstructure of the ReSe_2_ flake was detected by an atomic force microscope [(AFM), Cypher, Oxford Instruments] and a Raman spectrometer (Jovin Yvon T64000, excitation wavelength 532 nm). The AFM image of the ReSe_2_ flake is shown in Fig. 3a–c, and the determined average thickness is 28 nm based on the cross-sectional height profile along the white line. The Raman spectrum consisting of up to 13 expected lines with high signal strength is shown in Fig. 3d, corresponding well with the spectrum detected by Wolverson et al. [4] and revealing the triclinic crystal structure of the present ReSe_2_ flake.

**Fig. 4:**
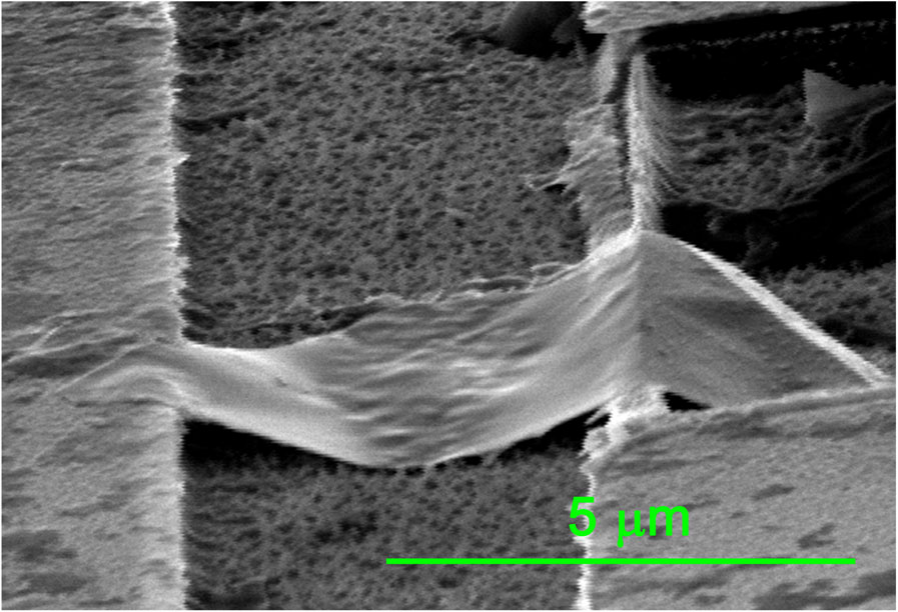
SEM image of the ReSe_2_ flake and the Au nanofilm in slant view of 45°

**Fig. 5:**
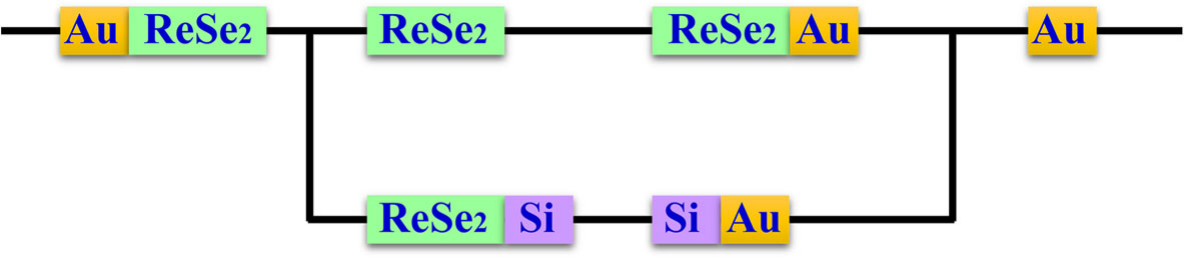
Schematic of the circuit

Figure 4 is the SEM image of the ReSe_2_ flake in slant view of 45° showing that the ReSe_2_ flake and the Au nanofilm are in contact with the Si substrate. ReSe_2_-Au contact has been shown the Ohmic contact in previous study [20] which is not responsible for the rectification behavior in this experiment. The circuit is constituted of the Au-ReSe_2_-Au and the Au-ReSe_2_-Si-Au junctions. Figure 5 shows the schematic of the circuit. The Si-Au contact has been shown the Schottky contact [21].

**Fig. 6:**
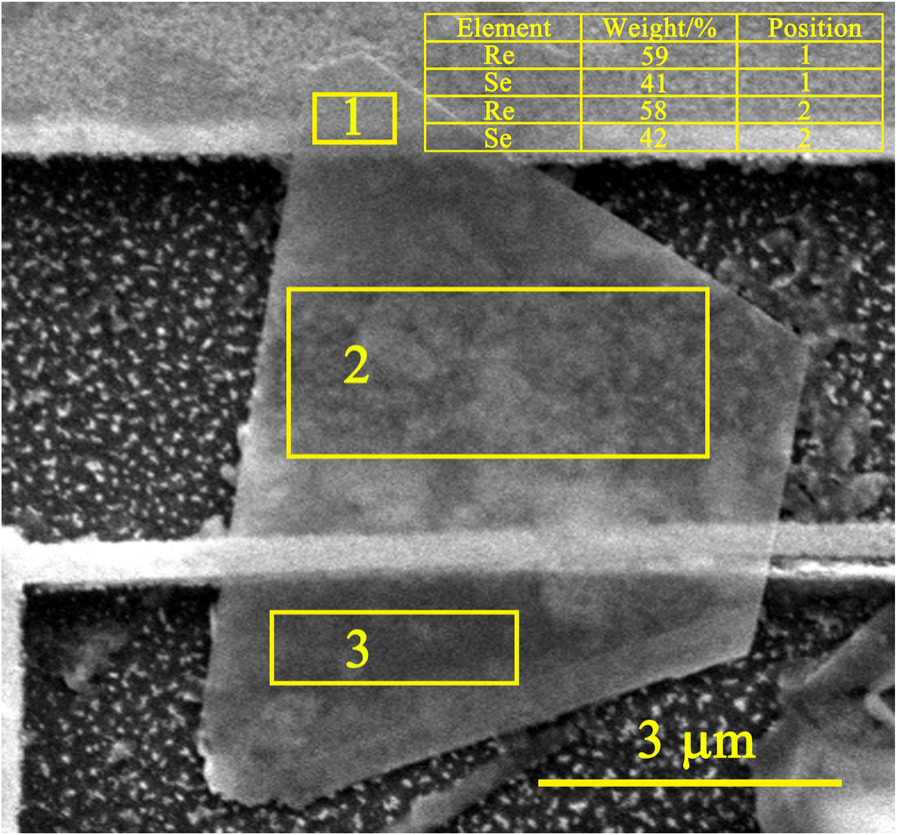
EDS data of ReSe_2_ is on the top right corner of the image. Boxes 1 and 2 represent two measured sections

Figure 6 shows the energy dispersive spectroscopy (EDS) data. The map sum spectrum of ReSe_2_ is acquired in section 1 and 2. The average chemical formula is ReSe_1.67_ which has a higher ratio of Re than ReSe_2_ and gives the ReSe_2_ flake p-type semiconductor properties. Therefore, the ReSe_2_-Si contact is a p-n heterojunction and exhibits the rectification behavior. Asymmetry of both rectification contacts results in the rectification behavior.

The current can be determined by the following equation in both the Schottky contact and the p-n heterojunction [22, 23]:

1 $$ I={I}_0{e}^{qV/ nkT}\left(1-{e}^{- qV/ kT}\right) $$

2 $$ {I}_0={AA}^{\ast }{T}^2{e}^{-q{\Phi}_B/ kT} $$

where *I*_0_ is the saturation current, *q* is the electronic charge, *k* is the Boltzmann constant, *V* is voltage applied across the junction, *A* is the contact area, A^*^ is the effective Richardson constant, *Ф*_B_ is the apparent barrier height, and *T* is the measurement temperature. The temperature-dependent ideality factor *n* represents the level that the contact departs from an ideal Schottky contact.

A calculation based on Eq. (1) is made to examine the analysis for the rectification behavior. Currents of the ReSe_2_-Si contact, *I*_1_, and the Si-Au contact, *I*_2_, are expressed by:

3 $$ {I}_1={I}_{01}{e}^{qV/{n}_1 kT}\left(1-{e}^{- qV/ kT}\right), $$

4 $$ {I}_2={I}_{02}{e}^{- qV/{n}_2 kT}\left({e}^{qV/ kT}-1\right). $$

**Fig. 7:**
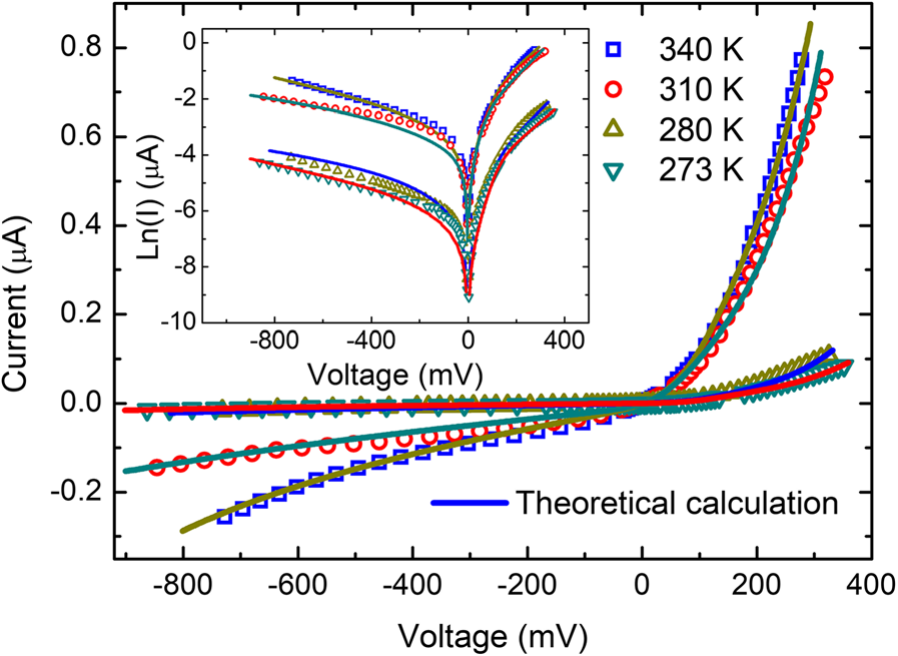
Comparison of *I*-*V* curves of the experimental results and the calculated

Figure 7 shows that the numerical results agree well with experimental data. The numerical parameters are shown in Table 1. The reverse saturation current of the ReSe_2_-Si contact is larger than the Si-Au contact because the contact area of the ReSe_2_-Si contact is much larger as shown in Fig. 4. The reverse saturation current of both contacts increase with temperature, indicating that the electrical conductivities of both contacts exhibit rectification behavior as shown in Eq. (2).

**Table 1:** Calculated ideality factor for ReSe_2_-Si and Si-Au contacts

Temperature/K	*I*_01_/μA	*I*_02_/μA	*n*_1_	*n*_2_
273	0.0095	0.0002	1.43	1.10
280	0.014	0.0003	1.46	1.08
310	0.084	0.011	1.46	1.06
340	0.170	0.010	1.46	1.11

The ideality factor of the ReSe_2_-Si contact is larger than the Si-Au contact due to different contact conditions and crystal structures. Figure 4 shows that the surface of the Si substrate is rough due to the etching solution, which makes the ReSe_2_-Si contact inhomogeneous. The inhomogeneous contact leads to the large ideality factor [24, 25]. The rough surface also produces a large number of trapping states which results in a large ideality factor [26]. Additionally, different contact types make different ideality factors. The ReSe_2_-Si contact is the p-n heterojunction, and the ReSe_2_ and Si have different crystal structures, triclinic for ReSe_2_ and faced-centered cubic for Si. The lattice mismatch always leads to edge dislocation [27] and produces high density of trap states [26], making the ReSe_2_-Si contact deviate from the ideal contact and have a large ideality factor [27]. The Si-Au is the metal semiconductor contact, and the crystal structure of Si has few effects on the ideality factor. The ideality factors of both contacts change little with temperature. It can be explained by Eq. (5) as reported by Khurelbaatar et al. [28],

5 $$ n=\frac{q}{kT}\frac{dV}{d\ln I}. $$

Equation (5) shows that the ideality factor is inversely proportional to the temperature. The ideality factor significantly decreases with temperature only at low temperature and changes slowly when the temperature is over 300 K [28, 29]. However, as shown in Table 1, the reverse saturation current increases significantly with the temperature which is different from the ideality factor. It can be explained by Eq. (2). According to Eq. (2), the reverse saturation current increases with temperature because *T*^2^ and exp (− *q*Φ_*B*_/*kT*) increase with temperature. Due to the exponential relationship between exp (− *q*Φ_*B*_/*kT*) and − qΦ_B_/kT, exp (− *q*Φ_*B*_/*kT*) increases significantly with temperature. Based on the research by Zhu et al [30], *q*Φ_*B*_ of the Au/Si contact in the experiment at 273 K and 295 K are 0.77 eV and 0.79 eV, respectively. The calculated results show that the reverse saturation current at 295 K is six times as much as the reverse saturation current at 273 K, explaining why the reverse saturation current increases significantly with temperature.

## Conclusions

In conclusion, a rectification behavior is observed in the contacts where a ReSe_2_ flake suspended across Au substrate and Au nanofilm at different temperature. The SEM image of the suspended ReSe_2_ flake in slant view of 45° shows that the ReSe_2_ flake and the Au nanofilm are in contact with the Si substrate and the EDS map illustrated the elements composition, ReSe_1.67_. The contact between the ReSe_2_ flake and the Si substrate is responsible for the rectification behavior. The ReSe_2_-Si and Si-Au contacts are both rectification contacts forming another circuit, and asymmetry of both contacts results in the apparent rectification behavior. The calculated results based on Schottky current equation considered the Si-Au Schottky contact, and the ReSe_2_-Si p-n heterojunction agrees well with experiments results.

## Abbreviations

2D: Two-dimensional
AFM: Atomic force microscope
EDS: Energy dispersive spectroscopy
FET: Field effect transistor
PMMA: Polymethylmethacrylate
SEM: Scanning electron microscope
TMD: Transition metal dichalcogenides
